# Changes in Membrane Cholesterol Differentially Influence Preferential and Non-preferential Signaling of the M1 and M3 Muscarinic Acetylcholine Receptors

**DOI:** 10.1007/s11064-014-1325-z

**Published:** 2014-05-13

**Authors:** Pavel Michal, Esam E. El-Fakahany, Vladimír Doležal

**Affiliations:** 1 Institute of Physiology, Academy of Sciences of the Czech Republic, v.v.i., Vídeňská 1083, 14220 Prague, Czech Republic; 2 Department of Experimental and Clinical Pharmacology, University of Minnesota College of Pharmacy, Minneapolis, MN 55455 USA

**Keywords:** Muscarinic receptors, Agonist binding, Cholesterol, G-Proteins, Signal transduction, cAMP synthesis

## Abstract

We have found earlier that changes in membrane cholesterol content have distinct impact on signaling via the M1, M2, or M3 receptors expressed in CHO cells (CHO-M1 through CHO-M3). Now we investigated whether gradual changes in membrane cholesterol exerts differential effects on coupling of the M1 and M3 muscarinic receptors to preferential signaling pathways through G_q/11_ and non-preferential G_s_ G-proteins signaling. Changes in membrane cholesterol resulted in only marginal alterations of antagonist and agonist affinity of the M1 and M3 receptors, and did not influence precoupling of either subtype. Changes in membrane cholesterol did not influence parameters of carbachol-stimulated GTP-γ^35^S binding in CHO-M1 membranes while reduction as well as augmentation of membrane cholesterol lowered the efficacy but increased the potency of carbachol in CHO-M3 membranes. Gradual increase or decrease in membrane cholesterol concentration dependently attenuated agonist-induced inositolphosphates release while only cholesterol depletion increased basal values in both cell lines. Similarly, membrane cholesterol manipulation modified basal and agonist-stimulated cAMP synthesis via G_s_ in the same way in both cell lines. These results demonstrate that changes in membrane cholesterol concentration differentially impact preferential and non-preferential M1 and M3 receptor signaling. They point to the activated G-protein/effector protein interaction as the main site of action in alterations of M1 receptor-mediated stimulation of second messenger pathways. On the other hand, modifications in agonist-stimulated GTP-γ^35^S binding in CHO-M3 membranes indicate that in this case changes in ligand-activated receptor/G-protein interaction may also play a role.

## Introduction

Muscarinic receptors belong to the family of G-protein coupled receptors (GPCR) that are the most abundant and pharmacologically targeted plasma membrane receptors [9, 22]. A common structural feature of GPCR is the extracellular N-terminus, seven membrane spanning domains, three extracellular, three intracellular loops, and intracellular C-terminus. Stimulation of GPCR leads to activation of specific G-proteins and their intracellular signaling pathways that play important regulatory roles in virtually all physiological functions. There are five subtypes of muscarinic receptors denoted as M1-M5 and encoded by five different genes [2–5, 31]. Individual muscarinic receptor subtypes share a high degree of homology in the transmembrane domains while the extracellular and intracellular loops are less conserved [13–15]. The intracellular C-terminus may form the fourth intracellular loop by means of a glycosyl anchor. The N-terminal part of the third intracellular loop represents the contact domain for interaction with G-proteins [12, 37]. Higher variability of this domain enables selectivity of interaction with different G-proteins.

The M1, M3, and M5 receptor subtypes preferentially activate Gq/11 G-protein intracellular signaling while the M2 and M4 subtypes prefer Gi/o G-proteins and activate their signaling pathways [20, 21]. However, it has been demonstrated that individual subtypes of muscarinic receptors can also interact with and activate non-preferential G-proteins [17, 19, 26, 27]. We have previously reported that experimental changes in membrane cholesterol concentration have various impacts on preferential second messenger signaling mediated by M1, M2, and M3 muscarinic receptors [28]. With respect to M1 and M3 receptors that prefer G_q/11_ G-proteins we found that both increase and decrease in membrane cholesterol attenuates maximal effect (efficacy) of carbachol, a non-hydrolysable analog of the natural agonist acetylcholine, in stimulating inositolphosphates (IPs) accumulation but does not influence its potency. In contrast, an increase in membrane cholesterol had no influence on preferential M2 receptor mediated inhibition of cAMP synthesis while cholesterol depletion increased inhibition by carbachol without influencing its potency.

However, as mentioned above, odd numbered muscarinic receptors expressed in CHO cells also activate G_s_ G-proteins. There can be mechanistic differences between G_q/11_ and G_s_ G-protein activation of intracellular signaling pathways mediated by M1 or M3 receptors. While these receptors expressed in CHO cells precouple with G_q/11_ and G_i/o_ G-proteins they do not precouple with G_s_ G-proteins [19]. In the present experiments we explored the influence of gradual changes in membrane cholesterol concentration on individual steps of signal transduction via M1 muscarinic receptors, including agonist binding, activation of G-proteins, and resulting stimulation of intracellular signaling pathways. Our aim was to reveal if there are differences between the effects of changing membrane cholesterol content on G_q/11_ and G_s_ G-proteins signaling. To achieve this goal we determined binding parameters and functional response in CHO cells expressing the muscarinic M1 receptor (CHO-M1 cells) that prevails in the brain. For comparison we used CHO cells that express the M3 muscarinic receptor (CHO-M3). We demonstrate that changes in membrane cholesterol do not influence precoupling of either M1 or M3 receptors and result in only marginal alterations of antagonist and agonist affinity. Despite only slight alterations of receptor/G-protein interactions both increase and decrease in membrane cholesterol evoked significant modifications in cAMP synthesis.

## Methods

### Cell Lines, Treatments, and Chemicals

Experiments were performed essentially as described previously in Michal et al., 2009 [28]. Briefly, CHO cells stably transfected with the human genes of the muscarinic M_1_ and M_3_ receptor subtypes (CHO-M1 and CHO-M3 cells, respectively) were kindly supplied by Prof. Tom Bonner. Cells were grown in Dulbecco’s modified Eagle’s medium (DMEM) with 10 % fetal calf serum and 0.005 % geneticin, and used for experiments three to 5 days after seeding. Cells were grown in 10 cm diameter Petri dishes for preparation of membranes or in 24-well or 48-well plates for assays on intact cells. Before experiment, cells for functional assays were loaded with ^3^H-adenine for 4 h or with ^3^H-myo-inositol for 4–12 h in DMEM. Then they were treated for 1 h at 37 °C in DMEM with indicated concentrations of methyl-β-cyclodextrin (MBCD; ranging from 1.25 to 10 mM) to deplete membrane cholesterol or with cholesterol-saturated methyl-β-cyclodextrin (Ch-MBCD; ranging from 0.25 to 4 mM) to increase membrane cholesterol. Cholesterol-modifying medium was washed off using DMEM and cells were used for measurements on intact attached cells or for preparation of membranes for binding assays.

Chemicals were obtained from Sigma (Prague, Czech Republic) unless indicated otherwise.

### Membrane Preparation

Membranes were prepared as described by Jakubík et al. [17] from control cells or from cells that had been treated with cholesterol-modifying agents as described above.

### Saturation and Competition Binding Assays

Binding characteristics of muscarinic receptors were determined in equilibrium binding experiments with the membrane-impermeable quaternary amine muscarinic antagonist [^3^H]-*N*-methylscopolamine (^3^H-NMS) (ARC, USA) as a tracer. Densities and affinities of muscarinic receptors were determined in saturation assays on intact attached cells or cell membranes as described [17, 18, 33].

Intact cells were incubated for 1 h at 37 °C in 0.5 ml of DMEM containing increasing concentrations of ^3^H-NMS ranging from 63 pM to 2.0 nM. Attached cells were then quickly washed with cold phosphate buffered saline to remove unbound ligand, dissolved in 1 M NaOH, and aliquots of these lysates were used for scintillation counting and protein determination. Membranes were suspended in binding buffer (100 mM NaCl, 10 mM MgCl_2_, 20 mM Na-HEPES, pH 7.4) and aliquots (5–20 μg of protein in a final volume 400 μl) in 96-well-plate were incubated for 1 h at 25 °C in the presence of 63 pM–2 nM ^3^H-NMS. Incubation was terminated by filtration through Whatman GF/B glass fiber filters (Whatman) using a Brandel harvestor (Brandel, USA). Filters were dried and counted in scintillation cocktail Rotiszint (Carl Roth, Germany) in Wallac Microbeta scintillation counter (Wallac, Finland). Non-specific binding was determined in the presence of 10 μM atropine.

Binding characteristics of the muscarinic agonist carbachol were determined analogically in competition experiments with 1 nM [^3^H]-NMS as a tracer.

### Functional Assays

IPs accumulation and cAMP production were assayed in attached cells grown in 24-well-plates as described [28]. For cAMP synthesis measurements, [^3^H]-adenine (10 μCi/ml; GE Healthcare, UK) labeled control or treated cells were preincubated in DMEM containing 1 mM isobutylmethylxanthine for 15 min and then in the presence of increasing concentrations of carbachol for 10 min at 37 °C. The reaction was stopped by adding trichloroacetic acid (TCA) and cyclic [^14^C]-AMP (GE Healthcare, UK) that was used as recovery standard. Aliquots of TCA extracts were used for determination of TCA-soluble radioactivity and separation of [^3^H]-AMP from other labeled metabolites [16, 27]. TCA precipitates were dissolved in 1 M NaOH and used for determination of protein content.

For IPs accumulation, [^3^H]myo-inositol (10 μCi/ml; GE Healthcare, UK) labeled control or treated cells were preincubated in DMEM containing 12 mM LiCl for 15 min and then in the presence of increasing concentrations of carbachol for 10 min at 37 °C. The incubation was stopped on ice by adding TCA. Accumulated TCA-soluble radioactivity was used for estimation of formation of inositol phosphates without further separation [28]. TCA precipitates were dissolved in 1 M NaOH and aliquots of these lysates were used for scintillation counting for determination of radioactivity loading and protein content determination.

Activation of G-proteins by agonist reflects signal transduction across plasma membrane. Muscarinic receptor-induced activation of G-proteins was determined as an increase of GTP-γ^35^S binding to membranes induced by the muscarinic receptor agonist carbachol as described [17, 24]. Aliquots of membranes containing 5–20 μg protein were preincubated in 96-well-plate at 30 °C in binding buffer containing in addition 1 mM DTT, 1 μM GDP, and concentrations of carbachol ranging from 0 to 100 μM. Reaction was started by adding GTP-γ^35^S (Biotrend Chemikalien, Germany; SRA 1,000 Ci/mmol) to give a final concentration of 500 pM and incubation continued for another 20 min. Total content of G-proteins in membranes was determined as GTP-γ^35^S binding in the absence of GDP. Nonspecific binding was assessed in the presence of 10 μM unlabeled GTP. Incubation was terminated by rapid vacuum filtration through Whatman GF/F filters using Tomtec Harvester Mach III (USA). Radioactivity retained on filters was determined using solid scintillator Meltilex (Perkin Elmer, USA) as described for [^3^H]-NMS binding to membranes.

### Data Evaluation

Curve fitting and statistical evaluation of data was done using Prism 6 (GraphPad Software Inc., CA, USA). Rectangular hyperbola was fitted to data obtained in saturation analysis experiments. A sigmoidal concentration–response curve equation with constant or variable slope as appropriate was fitted to data obtained in GTP-γ^35^S binding and cAMP synthesis experiments. A two-sites displacement curve equation was fitted to data obtained in displacement experiments. Better fits were determined using *F* test. The significance of differences among groups was tested by Anova and indicated post hoc test or *t* test as appropriate. Results are shown as mean ± SEM.

## Results

We did not find differences between CHO-M1 and CHO-M3 cells in cell or membrane cholesterol concentration, either in control values or values after treatment with MBCD or Ch-MBCD. These values were therefore pooled (Table 1). Changes in cell and membrane cholesterol concentration were proportional.

**Table 1:** Influence of changes in cholesterol concentration on ^3^H-*N*-methylscopolamine binding in intact CHO-M1 and CHO-M3 cells

Treatment	MBCD (10 mM)	MBCD (5 mM)	Control	Ch-MBCD (2 mM)	Ch-MBCD (4 mM)
Cell cholesterol (nmol/mg protein)	13.1 ± 0.9 (6)	24.5 ± 1.1 (6)	51.8 ± 3.0 (6)	123.7 ± 11.9 (6)	178.5 ± 13.7 (6)
Membrane cholesterol (nmol/mg protein)	61.6 ± 3.2 (6)	108.6 ± 5.5 (6)	212.2 ± 11.4 (6)	555.8 ± 38.5 (6)	745.7 ± 55.9 (6)
CHO-M1 cells					
B_max_ (pmol/mg protein)	8.58 ± 0.30** (3)	5.97 ± 0.05** (3)	3.59 ± 0.06 (3)	3.12 ± 0.20 (3)	2.36 ± 0.22** (3)
K_d_ (pM)	454.7 ± 11.7* (3)	308.4 ± 6.4 (3)	259.1 ± 2.6 (3)	358.2 ± 60.4 (3)	370.8 ± 52.7 (3)
CHO-M3 cells					
B_max_ (pmol/mg protein)	3.82 +/0.14** (3)	3.20 ± 0.12** (3)	2.40 ± 0.11 (3)	2.22 ± 0.14 (3)	1.35 ± 0.20** (3)
K_d_ (pM)	289.4 ± 16.2 (3)	257.2 ± 8.9 (3)	244.5 ± 10.0 (3)	238.5 ± 6.9 (3)	396.5 ± 14.2** (3)

Data are expressed as mean ± SEM of n experiments (in parentheses). Values for cholesterol concentrations were pooled because they did not differ between CHO-M1 and CHO-M3 cells and membranes. * *p* < 0.05; ** *p* < 0.01; significantly different from controls (middle column) by Anova followed by Dunnett’s multiple comparison test

**Fig. 1:**
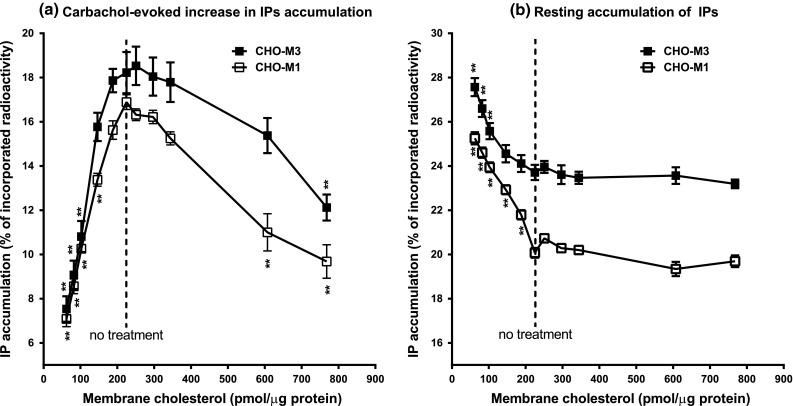
Influence of membrane cholesterol concentration on efficacy of signal transduction in CHO-M1 and CHO-M3 cells. IPs accumulation in intact CHO cells expressing M1 (*open squares*) or M3 (*closed squares*) receptors induced by 100 μM carbachol was determined in cells pretreated with various concentrations of MBCD or cholesterol-saturated MBCD to decrease or increase membrane cholesterol content, respectively. Accumulation of IPs in the presence (**a**) or absence (**b**) of carbachol, expressed in percent of incorporated radioactivity (ordinate), is plotted against membrane cholesterol concentration (abscissa) determined after treatments. The cells were treated with 10–7.5–5–2.5–1.25 mM MBCD to reduce membrane cholesterol or with 0.25–0.5–1–2–4 mM Ch-MBCD to increase membrane cholesterol as described in “Methods” section. The corresponding averaged values of membrane cholesterol were 62, 82, 103, 146, 188, control 225, 251, 297, 344, 607, and 768 nmol/mg protein. Points are mean ± SEM of 3–4 experiments in triplicates. No treatment, control cells. **, *p* < 0.01 significantly different from control cells (*dotted line*) by Anova followed by Dunnett’s multiple comparison test

Treatment of CHO-M1 and CHO-M3 cells with 10, 7.5, 5, 2.5 or 1.25 mM MBCD or with 0.25, 0.5, 1, 2 or 4 mM Ch-MBCD resulted in expected concentration-dependent changes in membrane cholesterol concentration (see legend to Fig. 1). Changes in membrane cholesterol content (control cells 225 nmol/mg protein) ranged from a decrease induced by 10 mM MBCD to 62 nmol/mg protein up to an increase to 768 nmol/mg protein induced by 4 mM Ch-MBCD. The decrease as well as the increase in membrane cholesterol content concentration-dependently attenuated the efficacy of carbachol in stimulating IPs accumulation in both CHO-M1 and CHO-M3 cells (Fig. 1a). In contrast, decreasing membrane cholesterol concentration dependently increased resting IPs accumulation in both cell lines while an increase in membrane cholesterol had no effect (Fig. 1b). Nevertheless, CHO-M1 cells were somehow more sensitive to changes in membrane cholesterol concentration with respect to both resting and carbachol-evoked accumulation of IPs.

A decrease in membrane cholesterol augmented (by 139 and 59 % for M1 and M3 receptors, respectively; *p* < 0.01 for both subtypes) while an increase in membrane cholesterol lowered (to 66 and 56 % for M1 and M3 receptors, respectively; *p* < 0.01 for both subtypes) binding of the muscarinic receptor antagonist ^3^H-NMS in both cell lines. The affinity of ^3^H-NMS binding was only marginally affected by membrane cholesterol manipulation but, surprisingly, differed between receptor subtypes. Maximal cholesterol depletion decreased the affinity of ^3^H-NMS binding in CHO-M1 cells (from 259 to 455 pM; *p* < 0.05) but had no effect in CHO-M3 cells. Conversely, maximal supplementation of cholesterol decreased the affinity of ^3^H-NMS binding in CHO-M3 cells (from 245 to 397 pM; *p* < 0.01) but had no effect in CHO-M1 cells.

Changes in membrane concentration of cholesterol in CHO-M1 cells also had only marginal effects on agonist binding. In competition experiments summarized in Table 2 we used carbachol that is a non-hydrolysable analog of natural agonist acetylcholine and ^3^H-NMS as a tracer. Maximal cholesterol depletion induced by 10 mM MBCD significantly decreased the affinity of agonist low affinity binding in CHO-M1 cells (from 168 to 330 μM; *p* < 0.01) but had no effect on the proportion or affinity of high affinity binding sites. In contrast, it has no effect on the binding parameters of carbachol in CHO-M3 cells as well as in CHO-M1 and CHO-M3 membranes. Similarly, membrane cholesterol supplementation had no effect on the parameters of carbachol binding in either CHO-M1 or CHO-M3 cells or analogous membranes.

**Table 2:** Influence of changes in cholesterol concentration on carbachol binding in intact CHO-M1 and CHO-M3 cells and membranes

Treatment	MBCD (10 mM)	Control	Ch-MBCD (2 mM)
*M1 cells*			
Ki high (μM)	9.3 ± 3.9	5.6 ± 3.6	5.1 ± 3.6
Ki low (μM)	330 ± 34**	168 ± 23	239 ± 38
fH (%)	34.9 ± 5.0	28.4 ± 4.5	25.6 ± 4.9
(n)	(7)	(7)	(7)
*M3 cells*			
Ki high (μM)	4.7 ± 2.1	4.0 ± 3.0	4.1 ± 1.9
Ki low (μM)	87.0 ± 6.4	97.0 ± 17.7	84.8 ± 11.6
fH (%)	24.3 ± 5.5	12.3 ± 2.9	18.7 ± 1.5
(n)	(7)	(5)	(6)
*M1 membranes*			
Ki high (μM)	2.9 ± 1.1	3.3 ± 0.9	2.5 ± 0.2
Ki low (μM)	280 ± 47	233 ± 21	202 ± 21
fH (%)	29.9 ± 2.3	26.9 ± 2.1	28.8 ± 1.9
(n)	(5)	(6)	(5)
*M3 membranes*			
Ki high (μM)	1.42 ± 0.61	1.06 ± 0.56	0.40 ± 0.16
Ki low (μM)	80.4 ± 22.4	86.8 ± 31.3	53.4 ± 14.3
fH (%)	42.9 ± 3.3	45.8 ± 2.4	43.6 ± 2.7
(n)	(3)	(3)	(3)

Data are expressed as mean ± SEM of n independent experiments (in parentheses) in triplicates. Parameters of carbachol binding were calculated as described in “Methods”. ** *p* < 0.01; significantly different from controls (middle column) by Anova followed by Dunnett’s multiple comparison test

**Fig. 2:**
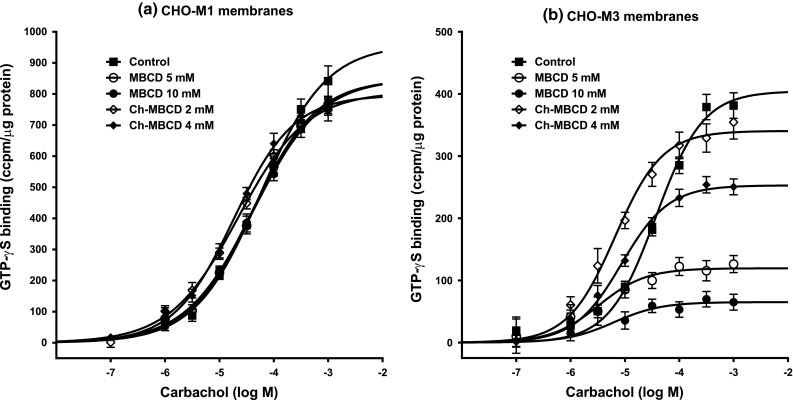
Influence of membrane cholesterol concentration on carbachol-evoked GTP-γ^35^S binding in CHO-M1 and CHO-M3 membranes. Membranes were prepared from cells pretreated with various concentrations of MBCD or cholesterol-saturated MBCD to decrease or increase membrane cholesterol content, respectively. Increase in GTP-γ^35^S binding in CHO-M1 (**a**) and CHO-M3 (**b**) membranes expressed in ccpm/μg protein (ccpm, corrected cpm) is plotted against carbachol concentration (abscissa; log M). The cells were treated with 10 or 5 mM MBCD (*closed* and *opened circles*, respectively) to reduce membrane cholesterol, or with 2 or 4 mM Ch-MBCD (*open diamond* and *closed diamonds*, respectively) to increase membrane cholesterol as described in “Methods”. Points are mean ± SEM of 2–3 experiments in triplicates or quadruplicates. *Closed squares*, control (DMEM treated) cells. Parameters of fits (four parameter sigmoidal equation for CHO-M1 cells and three parameters equation for CHO-M3 cells) are shown in Table 3

**Fig. 3:**
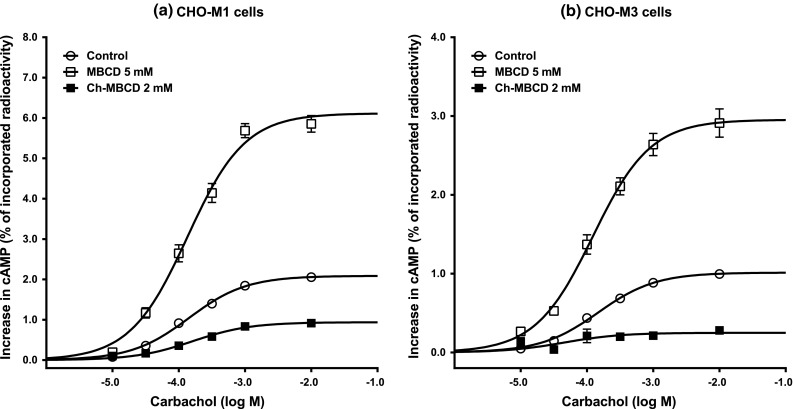
Influence of membrane cholesterol concentration on carbachol-evoked cAMP synthesis in CHO-M1 and CHO-M3 cells. Cells were labeled with ^3^H-adenine and then pretreated with MBCD or cholesterol-saturated MBCDto decrease or increase membrane cholesterol content, respectively. Increase in labeled cAMP synthesis in CHO-M1 (**a**) and CHO-M3 (**b**) cells expressed in percent of incorporated radioactivity is plotted against carbachol concentration (abscissa; log M). The cells were treated with 5 mM MBCD (*open squares*) to reduce membrane cholesterol or with 2 mM Ch-MBCD (*closed squares*) to increase membrane cholesterol as described in “Methods”. Points are mean ± SEM of 3 experiments in triplicates. *Open circles*, control (KHB treated) cells. Parameters of fits (three parameter sigmoidal concentration–response equation for both cell lines) are shown in Table 4

In the next two sets of experiments we probed the effects of changing membrane cholesterol content in functional tests characteristics of signal transduction across cell membrane and activation of the intracellular signaling pathways mediated by G_s_ G-proteins. First we tested carbachol-evoked stimulation of GTP-γ^35^S binding in membranes (Fig. 2). Total binding of GTP-γ^35^S (1 h incubation in the absence of GDP) that represents the level of activity of all available G-proteins incorporated in membranes did not differ between M1 and M3 membranes so the results were pooled (Table 3). Cholesterol depletion resulted in a small but significant increase of GTP-γ^35^S binding sites (by about 19 %; *p* < 0.01) while cholesterol supplementation slightly decreased GTP-γ^35^S binding (by about 8 %; *p* < 0.05). Resting binding of GTP-γ^35^S was significantly reduced in both CHO-M1 and CHO-M3 cholesterol-depleted membranes (by about 32 and 12 %, respectively; *p* < 0.01 for both subtypes) and slightly increased only in CHO-M1 cholesterol-supplemented membranes (by about 12 %; *p* < 0.05). Concentration–response relationship of carbachol-induced stimulation of GTP-γ^35^S binding (Fig. 2) in CHO-M1 membranes was best fitted by a four-parameter equation (with Hill slope) while that for CHO-M3 membranes by a regular sigmoidal concentration–response curve (three-parameter equation). Neither cholesterol depletion nor cholesterol supplementation changed the shape of the concentration–response curves. Changes in cholesterol of CHO-M1 membranes did not influence either maximal response evoked by carbachol (E_max_) or concentration of carbachol inducing half-maximal stimulation (EC_50_). In contrast, both elevation and depletion of membrane cholesterol of CHO-M3 membranes significantly increased potency of carbachol (*p* < 0.01) but attenuated maximal response (Table 3). Changes in parameters of basal and carbachol-evoked cAMP synthesis in intact CHO-M1 and CHO-M3 cells after cholesterol modifying treatments were similar for both cell lines (Fig. 3, Table 4). Depletion of membrane cholesterol increased basal and carbachol-evoked cAMP synthesis (*p* < 0.05 for both subtypes) while supplementation of membrane cholesterol had no effect on basal synthesis of cAMP but reduced cAMP synthesis evoked by carbachol (*p* < 0.05 for both subtypes). None of the treatments influenced potency of carbachol.

**Table 3:** Influence of changes in cholesterol concentration on carbachol-evoked GTP-γ^35^S in CHO-M1 and CHO-M3 membranes

Treatment	MBCD (10 mM)	MBCD (5 mM)	Control	Ch-MBCD (2 mM)	Ch-MBCD (4 mM)
*Pooled M1 and M3*					
Total binding (ccpm/μg)	5,310 ± 82**	4,987 ± 56**	4,469 ± 57	4,410 ± 63	4,127 ± 121*
(n)	(6)	(4)	(6)	(4)	(6)
*M1 membranes*					
Basal binding	1,004 ± 46**	997 ± 24**	1,473 ± 57	1,643 ± 32*	1,529 ± 30
E_max_ (ccpm/μg)	854 ± 31	857 ± 39	974 ± 11	810 ± 4	819 ± 81
EC_50_ (μM)	42.5 ± 4.0	44.4 ± 11.8	64.6 ± 16.8	22.4 ± 0.9	18.5 ± 0.2
Hill slope	0.708 ± 0.052	0.745 ± 0.119	0.720 ± 0.066	0.679 ± 0.009	0.758 ± 0.059
(n)	(3)	(2)	(3)	(2)	(3)
*M3 membranes*					
Basal binding	1,096 ± 30**	1,172 ± 18	1,248 ± 27	1,333 ± 34	1,278 ± 17
E_max_ (ccpm/μg)	77.0 ± 11.0**	118.5 ± 0.5**	401 ± 30.8	341.5 ± 43.5	254.7 ± 18.9*
EC_50_ (μM)	2.7 ± 1.0**	3.1 ± 2.1**	36.9 ± 2.6	6.6 ± 0.2**	9.3 ± 1.8**
Hill slope	1	1	1	1	1
(n)	(3)	(2)	(3)	(2)	(3)

Data are expressed as mean ± SEM of n experiments (in parentheses) in triplicates or quadruplicates. Values for GTP-γ^35^S binding are expresse in ccpm/μg protein. Values for total binding in CHO-M1 and CHO-M3 membranes did not differ so they were pooled. * *p* < 0.05; ** *p* < 0.01; significantly different from controls (middle column) by Anova followed by Dunnett’s multiple comparison test

**Table 4:** Influence of changes in cholesterol concentration on carbachol-evoked cAMP synthesis in CHO-M1 and CHO-M3 cells

Treatment	MBCD (5 mM)	Control	Ch-MBCD (2 mM)
*M1 cells*			
Basal synthesis (%)	0.97 ± 0.13**	0.45 ± 0.02	0.40 ± 0.02
E_max_ (%)	6.13 ± 0.32**	2.09 ± 0.08	0.94 ± 0.07*
EC_50_ (μM)	143 ± 23	146 ± 22	170 ± 29
*M3 cells*			
Basal synthesis (%)	0.98 ± 0.12*	0.59 ± 0.09	0.56 ± 0.04
E_max_ (%)	2.95 ± 0.32**	1.01 ± 0.03	0.25 ± 0.07*
EC_50_ (μM)	124 ± 4	147 ± 27	110 ± 93

Data are expressed as mean ± SEM of three independent experiments in triplicates. Values for cAMP synthesis are expressed in percent of loaded radioactivity that did not differ among treatments. Pooled value of incorporated radioactivity was 216,550 ± 10,542 dpm/well (n = 18). * *p* < 0.05; ** *p* < 0.01; significantly different from controls (middle column) by Anova followed by Dunnett’s multiple comparison test

## Discussion

The M1 muscarinic receptor is a major cerebral muscarinic receptor subtype that is essential for cognitive functions. Any malfunction of its G_q/11_ G-protein-mediated signaling may thus adversely impact not only mental performance but also amyloid precursor protein processing and amyloid-β generation [6, 29, 30]. We probed the influence of changing membrane cholesterol concentration on M1 and M3 receptor activation and signaling. Our results indicate that both an increase and a decrease in membrane cholesterol concentration-dependently attenuate maximal stimulation of preferential M1 and M3 receptor-mediated G_q/11_ G-protein signaling. Moreover, depletion of membrane cholesterol has more pronounced effects than cholesterol supplementation on the efficacy of the agonist carbachol. This observation is in line with our preceding finding of reduced efficacy of carbachol-stimulated IPs accumulation in CHO-M1 cells induced by membrane cholesterol manipulation [28].

With respect to ligand binding, the major outcome of cholesterol depletion common for both cell lines was an increase in the density of antagonist binding sites in both cell lines that was accompanied with a small decrease in affinity (by 71 %) only in CHO-M1 cells at the highest level of cholesterol reduction. Conversely, cholesterol supplementation decreased the density of binding sites at both cell lines only after the highest cholesterol supplementation with a small decrease in affinity (by 62 %) only in CHO-M3 cells. We observed similar effects of cholesterol depletion in CHO-M2 cells [28] so that we assume that changes in receptor densities after membrane cholesterol manipulations are due to modifications in the accessibility of receptors to the antagonist ligand consequent to alterations of membrane physicochemical properties [11]. Unlike in CHO-M2 cells and membranes, we found no essential membrane cholesterol concentration-dependent changes in parameters of agonist binding in CHO-M1 and CHO-M3 cell lines or membranes prepared from treated cells. The differential pattern of changes in ligand binding characteristics among these three cell lines indicate that they are not an experimental artifact arising out of the treatment.

Experiments aimed at determination of the effects of manipulation of membrane cholesterol on various elements of signal transduction yielded interesting results. There was no difference in total binding of GTP-γ^35^S (denoting G-protein concentration) between CHO-M1 and CHO-M3 (and also CHO-M2; not shown) membranes. Cholesterol depletion slightly augmented (by 18 %) while cholesterol supplementation slightly attenuated (by 8 %) total GTP-γ^35^S binding. We suppose that similar to antagonist binding, cholesterol concentration manipulations unmask or mask some GTP binding sites in membranes. In support of this view, western blot analysis of lysed membranes after cholesterol modifying treatment did not reveal any changes in the concentration of the major G-protein subclasses α-subunits indicating that the total protein concentration of α-subunits was not changed by the treatment [28]. Noteworthy, however, manipulation of cholesterol content may influence the stoichiomentry of GTP interaction with G proteins. While the influence of cholesterol modifications on resting (in the absence of agonist) binding was basically similar in both CHO-M1 and CHO-M3 membranes, carbachol-stimulated GTP-γ^35^S binding markedly differed between them. Agonist-stimulated GTP-γ^35^S binding in control CHO-M1 membranes was best fitted by a sigmoidal curve with Hill slope less than unity that was not at all influenced by changes in membrane cholesterol concentration. In contrast, carbachol-stimulated GTP-γ^35^S binding in CHO-M3 membranes followed a three-parameter sigmoidal concentration–response curve. In addition, unlike in CHO-M1 cells, both cholesterol depletion and supplementation concentration-dependently reduced the maximal effect of carbachol but increased its potency. Together with the lack of effect on agonist binding these results provide evidence for alteration of M3 receptor-mediated G_s_ G-protein signaling also upstream of activated G-protein/effector protein interaction.

Effects of changes in membrane cholesterol concentration exhibited different patterns on agonist-induced stimulation of the nonpreferential G_s_ G-protein signaling pathway than the pattern of overall G-protein activation or stimulation of the preferential phosphatidylinositol hydrolysis pathway. In both intact CHO-M1 and CHO-M3 cells, cholesterol depletion increased while cholesterol supplementation decreased carbachol-evoked cAMP synthesis. The sense of changes in muscarinic receptor stimulation-evoked metabolism of second messengers that result from alterations in membrane cholesterol concentration is basically the same in both cell lines. However, mechanisms of these changes differ in CHO-M1 and CHO-M3 cells. Results of binding experiments demonstrate that signal transmission across the membrane via the M1 receptor is not influenced by membrane cholesterol concentration. On the other hand, changes in membrane cholesterol concentration influence both agonist binding and G-protein activation in CHO-M3 cells. Together these data indicate that alterations in M1 receptor signaling as a results of changes in membrane cholesterol concentration are due to events downstream of receptor/G-protein activation while impact on M3 receptor signaling involves both components.

A role of cholesterol in the pathogenesis of Alzheimer’s disease remains a matter of controversy, in spite of the involvement of several research groups in such studies. Thus, considerable experimental evidence supporting both the beneficial and detrimental role of both increased and reduced cell cholesterol exists [25, 32, 36]. Most of experimental work has focused on mutual interactions of membrane cholesterol and constitutive cleavage of amyloid precursor protein and a role of β-amyloid in lipid metabolism [10]. It has been demonstrated that an increase in membrane cholesterol facilitates production of noxious β-amyloid fragments while reduction in membrane cholesterol has the opposite effect [7, 8, 34, 35, 38]. On the other hand, a decrease in membrane cholesterol concentration was detected in a subgroup of *post mortem* Alzheimer´s brains with reduced activity of the amyloid peptites degrading protease plasmin [23]. In other experiments, a small decrease in membrane cholesterol resulted in an increase in amyloid-β production while inhibition of amyloid-β production required a large decrease in cholesterol content [1]. Our results indicate that a relatively small drop in membrane cholesterol already results in highly significant attenuation in agonist-evoked G_q/11_ G-protein-mediated M1 receptor signaling (Fig. 1: decrease by 35 %, *p* < 0.0001 by Anova and Dunnett’s test; decrease by 17 %, *p* < 0.027 by two-tailed t-test) that may attenuate α-secretase activity.

In summary, we demonstrate that changes in membrane cholesterol concentration markedly influence M1 and M3 muscarinic receptor-mediated signaling to the cell interior. Our data illustrate that changes in membrane cholesterol concentration have essentially no influence on M1 receptor/G-proteins interactions, suggesting that changes in signaling take place distal to this step. With regard to the preferential G_q/11_ G-protein signaling pathway both increase and decrease in membrane cholesterol concentration results in concentration-dependent inhibition of accumulation of inositol phosphates. These effects are not due to a loss of phosphatidylinositol-specific phospholipase C because basal values (in the absence of agonist) are either not changed (high cholesterol) or increased (low cholesterol). Non-preferential agonist-stimulated G_s_ G-protein-mediated signaling differs from the inositol phosphates response. The decrease in membrane cholesterol concentration increases the cAMP response while increase in membrane cholesterol concentration inhibits the response. Taken together these results point to the activated G-protein/effector protein interaction as the main site of action in the observed alterations of M1 receptor-mediated stimulation of second messenger pathways. These changes most likely depend on physicochemical properties of the membrane and changes in receptor localization and mobility within the membrane. On the other hand, alterations in agonist-stimulated GTP-γ^35^S binding in CHO-M3 membranes indicate that in case of the M3 receptor additional modification of liganded receptor/G-protein interaction may also play a role.
